# 888. Open-access Outpatient Clinic Allows for Same Day Ambulatory Surgery for Walk-in Burn Patients

**DOI:** 10.1093/jbcr/irag033.374

**Published:** 2026-04-06

**Authors:** Emily Allred

**Affiliations:** Eastern Idaho Regional Medical Center Burn Center, Idaho Falls, ID

## Abstract

**Introduction:**

Due to the location of a rural burn center, walk-in burn patients are attracted from over 600mi (960 km) away. This distance creates a sizeable barrier if the patients have to return a different day for needed ambulatory surgery. An open-access outpatient clinic allows for walk-in burn patients to receive ambulatory surgery that same day.

**Methods:**

Open-access hours in the wound care clinic (WCC) were developed to allow for walk-in burn patients 7 days of the week. A “Burn Block” on 1 of the 14 operating rooms (ORs) in the hospital was developed to allow for priority and usage of the operating room for burn same-day surgeries (SDS) for walk-in burn patients. In order to determine impact of the open-access clinic, a retrospective study was ran on the number of SDS burn patients coming from the WCC. Burn patients were isolated from all walk-in patients to the WCC and then stratified as either not receiving SDS, SDS on same day as the first clinic visit, or SDS after multiple clinic visits or days.

**Results:**

Over the past three years, there has been a total of 752 walk-in burn patients in the open-access WCC. Of these patients, 366 (48.7%) of them received ambulatory SDS for their burn. Of the 366 patients, 242 (66.1%) of them received ambulatory same-day surgery the same day as their first WCC visit. The other 124 (33.9%) patients received ambulatory surgery after multiple clinic visits or days after their first WCC visit. When looking at all walk-in burn patients, patients receiving ambulatory SDS the same day as their first WCC visit is 32.2%.

**Conclusions:**

An open access outpatient clinic in conjunction with an OR burn block allows for walk-in burns to receive same day ambulatory surgery. Of all WCC walk-in patients with burns, 32.2% were able to have ambulatory surgery that same day. This removes a major barrier for patients traveling a far distance, eliminating the need for multiple visits prior to surgery. Notably, it also limits unnecessary admissions as a result.

**Applicability of Research to Practice:**

Open-access clinics and OR burn blocks eliminate the need for multiple clinic visits prior to initial surgery. This removes barriers for patients requiring treatment from long distance proximity to the burn center.

**Funding for the study:**

N/A.

